# *E. coli* Rep helicase and RecA recombinase unwind G4 DNA and are important for resistance to G4-stabilizing ligands

**DOI:** 10.1093/nar/gkaa442

**Published:** 2020-05-25

**Authors:** Tapas Paul, Andrew F Voter, Rachel R Cueny, Momčilo Gavrilov, Taekjip Ha, James L Keck, Sua Myong

**Affiliations:** Department of Biophysics, Johns Hopkins University, Baltimore, MD 21218, USA; Department of Biomolecular Chemistry, University of Wisconsin School of Medicine and Public Health, Madison, WI 53706, USA; Department of Biomolecular Chemistry, University of Wisconsin School of Medicine and Public Health, Madison, WI 53706, USA; Department of Biophysics, Johns Hopkins University, Baltimore, MD 21218, USA; Department of Biophysics, Johns Hopkins University, Baltimore, MD 21218, USA; Physics Frontier Center (Center for Physics of Living Cells), University of Illinois, 1110 W. Green St., Urbana, IL 61801, USA; Howard Hughes Medical Institute, Johns Hopkins University, USA; Department of Biomolecular Chemistry, University of Wisconsin School of Medicine and Public Health, Madison, WI 53706, USA; Department of Biophysics, Johns Hopkins University, Baltimore, MD 21218, USA; Physics Frontier Center (Center for Physics of Living Cells), University of Illinois, 1110 W. Green St., Urbana, IL 61801, USA

## Abstract

G-quadruplex (G4) DNA structures can form physical barriers within the genome that must be unwound to ensure cellular genomic integrity. Here, we report unanticipated roles for the *Escherichia coli* Rep helicase and RecA recombinase in tolerating toxicity induced by G4-stabilizing ligands *in vivo*. We demonstrate that Rep and Rep-X (an enhanced version of Rep) display G4 unwinding activities *in vitro* that are significantly higher than the closely related UvrD helicase. G4 unwinding mediated by Rep involves repetitive cycles of G4 unfolding and refolding fueled by ATP hydrolysis. Rep-X and Rep also dislodge G4-stabilizing ligands, in agreement with our *in vivo* G4-ligand sensitivity result. We further demonstrate that RecA filaments disrupt G4 structures and remove G4 ligands *in vitro*, consistent with its role in countering cellular toxicity of G4-stabilizing ligands. Together, our study reveals novel genome caretaking functions for Rep and RecA in resolving deleterious G4 structures.

## INTRODUCTION

Guanine-rich nucleic acid sequences have strong propensities to form four-stranded G-quadruplex (G4) structures under physiological conditions (1). In these structures, four guanine bases are cyclically coordinated through Hoogsteen hydrogen bonds to form a G-quartet or tetrad ring, which is further stabilized by stacking interaction with other ring layers in the presence of monovalent cation (1–3). G4s can fold into various conformations *in vitro* and evidence has confirmed that G4 structures are present in living cells (3,4). In the human genome, potential G4 clusters are enriched at important genomic regions including replication origins, oncogene promoters, telomeres, and immunoglobulin switch regions (5–7). G4 enrichment has also been found in *Escherichia coli* at regulatory regions of genes involved in transcription, secondary metabolite biosynthesis, and signal transduction (8–10). Inefficient regulation of G4 structures has been linked to genome instability (11). Stable G4 structures can act as roadblocks to numerous cellular processes such as replication, transcription, and translation (12–14). In addition, G4 ligands (*e.g*. BRACO-19 and N-methyl mesoporphyrin IX (NMM)) can further enhance the stability of G4 structures, disrupting critical cellular pathways and thereby inducing toxicity in cells (13,15). For example, BRACO-19 has been shown to inhibit DNA replication, transcription, and telomerase activity (15). Therefore, dedicated cellular machineries have evolved to resolve G4 structures.

Helicases are DNA unwinding motor proteins that play important roles in preserving genome integrity. Many helicases are capable of unwinding G4 DNA structures (16,17), including RecQ family helicases (bacterial RecQ, yeast Sgs1 and human BLM, WRN), XPD family enzymes such as FANCJ, and Pif1 family and DEAH box family including DHX36 (12,18–22). A recent study reported that a bacterial RecQ helicase, which possesses a guanine-specific binding pocket that is essential for G4 unwinding, resolves G4 through repetitive cycles of unwinding and refolding (23). In addition, several reports have shown that Pif1, BLM and DHX36 exhibit similar repetitive unfolding of G4 and can successfully dislodge G4-stabilizing ligands (20,24,25). In some cases, helicase activity was limited by G4 ligand binding (18,26).

The superfamily I helicases, UvrD and Rep are similar in structure and exhibit 3′-5′ direction of translocation, but do not overlap *in vivo* activity (27–30). UvrD plays an important role in nucleotide excision repair, mismatch repair and in the regulation of homologous recombination (31). Additionally, like the RecQ helicase, *E. coli* UvrD unwinds intermolecular and intramolecular G4 structures (31). Rep was the first helicase discovered in *E. coli* and unwinds DNA with a limited processivity of ≤400 bp (28,30,32). Rep shows low unwinding activity as a monomer *in vitro*, but multimerizes upon binding to DNA to show robust helicase activity (33). Intramolecular crosslinking of Rep monomer to Rep-X enhances the unwinding activity and makes Rep-X a processive superhelicase capable of continuous unwinding of more than 6000 base pairs without dissociation (34). Though Rep can unwind DNA, it is unclear whether Rep helicase can participate in the resolution of G4 structures.

In replication, recombinases assist in strand exchange repair for reloading of the accessory proteins. In fact, there is a significant interplay between accessory helicases and recombinases in both bacteria and lower eukaryotes (35). *Δrep* cells are still viable because UvrD partially compensates for the absence of Rep. However, the double deletion *Δrep* and *ΔuvrD* causes lethality (29,31,36). Furthermore, RecA and RecBCD can sustain viability in *Δrep* and *ΔuvrD* but only in the presence of an RNA polymerase mutation that alleviates transcriptional barriers to replication (35). *Escherichia coli ΔrecA* cells induce conflicts between replication and transcription, similar to the case of *Δrep* cells. Therefore, both RecA and Rep help mitigate the conflict between transcription and replication (35). *In vitro* study showed that *E. coli* RecA binds monomeric G4 from pilin expression locus (*pilE*) of *N. gonorrhoeae* with similar affinity to ss-DNA but does not bind other G4 structures (37). However, it still remains unclear whether RecA can resolve G4 structure.

To better define the mechanisms underlying G4 homeostasis in bacteria, we have identified genome maintenance genes in *E. coli* that are important for growth in the presence of G4-stabilizing ligands and show that each encodes a protein that is able to unwind G4 DNA structures *in vitro*. Δ*rep* and Δ*recA E. coli* strains are found to be sensitive to G4-stabilizing ligands whereas strains deficient in several other key genome maintenance genes are resistant to the compounds. Rep and RecA display robust G4 DNA unfolding and G4 ligand displacement activities *in vitro*. In contrast, UvrD, a helicase that shares significant structural similarity with Rep, demonstrated substantially weaker G4 unwinding activity. Rep translocates on single-stranded (ss) DNA in an ATP-hydrolysis dependent fashion and resolves G4 structures by repetitive cycles of unfolding activity. Rep-X, an enhanced version of Rep (34), displayed accelerated G4 unwinding activity via a similar mechanism. RecA disrupts G4 structures and dislodges G4 ligands by forming filaments along ssDNA. Together, the results suggest novel activities for Rep and RecA in resolving G4 structures that are important for protecting cells against the threat of G4 genomic roadblocks.

## MATERIALS AND METHODS

### Preparation of DNA constructs

The HPLC-purified DNA oligonucleotides (tabulated in Supplementary Table S1) containing both biotin for immobilization and either Cy3, Cy5 or amine modifications were purchased from IDT. Amine-modified oligonucleotides were labelled with NHS ester-conjugated fluorescent dyes following an established protocol (38). Briefly, 30 μl of 100 μM amine modified ssDNA was mixed and incubated overnight with 0.2 mg of NHS ester-conjugated Cy3 dye in 100 mM NaHCO_3_, pH 8.5. The excess dye was removed by ethanol precipitation and repeated twice. Each partial duplex DNA construct (10 μM) was prepared by mixing the biotin-conjugated DNA strand with its complementary strand at molar ratio of 1:1.2 (biotinylated:non-biotinylated) and annealed in T50 Buffer (10 mM Tris–HCl, pH 7.5 and 50 mM NaCl) in a thermocycler with the following protocol: 95°C for 2 min; gradual cooling to 40°C at the rate of 2°C/min; further cooling by 5°C/min until 4°C. G4-duplex (i.e. 42mer with T15) annealed in 10 mM Tris–HCl, pH 7.5 and 5 mM MgCl_2_ containing buffer following the same protocol as described above.

### Protein purification

*Escherichia coli* UvrD protein was purified as described previously (39). Briefly, UvrD was transformed into BL21(DE3) pLysS using kanamycin and chloramphenicol as selection markers. UvrD was overexpressed by adding 1 mM IPTG when the culture had an OD_600_ of 0.6; cells were then grown for an additional 4 hours at 37°C and subsequently pelleted and stored at -80°C. Cells were resuspended into Lysis Buffer (50 mM Tris pH 8.3, 10% sucrose, 200 mM NaCl, 5 mM ethylenediaminetetraacetic acid (EDTA), 0.5 mM ethylene glycol-bis (β-aminoethyl ether)-*N*,*N*,*N*’,*N*’-tetraacetic acid (EGTA), 15 mM 2-mercaptoethanol, 0.1 mM phenylmethylsulfonyl fluoride, 100 ug/ml lysozyme, and one Roche protease inhibitor tablet), lysed by sonication, and centrifuged at 34 864 × g. Nucleic acid contaminants were removed from the supernatant by the addition of Polymin P to a final concentration of 0.3% (v/v) followed by centrifugation at 34 864 × g. UvrD was precipitated from the supernatant by adding ammonium sulfate to a final concentration of 176 g/l and centrifuged again at 34 864 × g. The resulting pellet was resuspended into Resuspension Buffer (20 mM Tris pH 8.3, 20% glycerol, 1 mM EDTA, 0.5 mM EGTA, 15 mM 2-mercaptoethanol, 300 mM NaCl) followed by another centrifugation step at 34 864 × g. The resulting supernatant was mixed 1:2 with Buffer A (20 mM Tris pH 8.3, 20% glycerol, 1 mM EDTA, 0.5 mM EGTA, 15 mM 2-mercaptoethanol) and loaded onto a HiPrep Heparin FF affinity column (GE Healthcare). The column was washed thoroughly with Buffer A followed by protein elution using a gradient of 0.1 to 0.45 M NaCl in Buffer A. UvrD-containing fractions were collected and concentrated into ∼2 ml and loaded onto a HiPrep 16/60 Sephacryl S-300 column (GE Healthcare) that was equilibrated previously with Buffer B (20 mM Tris pH 8.3, 20% glycerol, 1 mM EDTA, 0.5 mM EGTA, 15 mM 2-mercaptoethanol, 500 mM NaCl). UvrD-containing fractions were concentrated, dialyzed into storage buffer (20 mM Tris pH 8.3, 50% glycerol, 1 mM EDTA, 0.5 mM EGTA, 25 mM 2-mercaptoethanol, 200 mM NaCl), and stored at –20°C.

*Escherichia coli* Rep was purified as described previously (40). Briefly, pET28a(+) vector containing Rep-DM4 was transformed into *E. coli*. BL21(DE3), and cells were induced at OD_600_ of 0.6 with 0.5 mM IPTG and harvested after an overnight incubation at 18°C. Cell pellets were resuspended in lysis buffer and sonicated followed by centrifugation at 34 864 × g. N-terminally 6xHis-tagged Rep protein was purified using Ni-NTA column and eluted with 150 mM imidazole containing buffer. The protein concentration was always kept below 4 mg/ml (50 μM) to avoid aggregation, and the final Rep protein was stored at –20°C with 50% glycerol.

Rep crosslinking (Rep-X) was performed using 10 mM BMOE (bismaleimidoethane) crosslinker solution in DMF (34). Optimal crosslinking was achieved at concentration of 20–25 μM and the final molar ratio of Rep and BMOE was 1:5. Excess imidazole and crosslinker were removed by overnight dialysis in 600 mM NaCl. Rep-X was finally stored at –20°C in storage buffer (50% glycerol, 600 mM NaCl, 50 mM Tris, pH 7.6).

### *E. coli* strain construction

*Escherichia coli* knockout strains were generated using P1 transductions as described previously (41). Briefly, P1 phage lysate was grown on each donor knockout strain and used to transduce the kanamycin-sensitive parent *MG1655* or *imp4213* strains. Individual knockout strains were gifts from Michael Cox. Kanamycin resistant colonies were isolated and insertion of the kan cassette was confirmed by PCR.

### Sensitization to G4 stabilizing ligands

NMM and BRACO-19 were resuspended in 18 MΩ ultrapure water, the concentration of NMM was measured using molar extinction coefficient as 145 000 M^−1^ cm^−1^ at 379 nm as described previously (42), and the resuspended solutions were stored at 4°C. The G4 stabilizing ligand was added to molten LB-agar to the specified concentration during plating and plates were stored in dark at 4°C. Cultures of each knockout strain were grown overnight at 37°C in 5 ml of LB supplemented with 50 ug/ml kanamycin. Next, overnight cultures were diluted to OD_600_ ∼ 1. The cultures were then serially diluted from 10^−1^ to 10^−6^ in LB, and 10 μl of the indicated dilution was plated onto prewarmed LB-agar plates with or without G4 stabilizer. These plates were grown overnight at 37°C and imaged.

### Single-molecule FRET assays and data acquisition

Single-molecule FRET (smFRET) data were acquired using a custom-built prism-type total internal reflection (PTIR) inverted fluorescence microscope (Olympus IX 71) as described previously (25,43,44). All experiments were carried out on quartz slides and coated with polyethylene glycol (PEG) to avoid non-specific interactions of excess DNA and protein. First, the predrilled quartz slides and glass coverslips were washed thoroughly with methanol, acetone, and etched by sonication in 1 M potassium hydroxide. Then slides were burned for 2–3 min, and coverslips were quickly sterilized by passing through the flame 4–5 times to remove all sources of fluorescence. Subsequently, both slides and coverslips were treated with aminosilane for 30 min and finally coated with a mixture of 98% mPEG (m-PEG-5000; Laysan Bio) and 2% biotin PEG (biotin-PEG-5000; Laysan Bio). The microfluidic sample chamber was created between the plasma-cleaned slide and coverslip coated with PEG and biotin-PEG.

Stocks of annealed partial duplex DNA (in T50 buffer) labelled with biotin, Cy3, and Cy5 were diluted to 15–20 pM using buffer consisting of 10 mM Tris–HCl, pH 7.5 and 100 mM KCl (to ensure stable G-quadruplex formation). Diluted DNAs were immobilized on the PEG-passivated surface via the biotin–neutravidin (50 μg/ml) interaction and unbound molecules were washed out by flowing excess buffer. All smFRET measurements were carried out in an imaging buffer containing 10 mM Tris–HCl, pH 7.5, 50 mM KCl, 3 mM MgCl_2_, 10% glycerol, and an oxygen scavenging system (10 mM Trolox, 0.5% (w/v) glucose, 1 mg/ml glucose oxidase and 4 μg/ml catalase) to avoid blinking and improve dye stability. Milli-Q water was used to prepare all buffers and then filtered through 0.22 μm membrane filters. All experimental data were recorded at room temperature (∼23°C ± 2°C).

A solid-state 532 nm diode laser (Compass 315M, Coherent) was used to generate an evanescent field of illumination to excite the Cy3 dye (donor) and the fluorescence from Cy3 and Cy5 (acceptor) were simultaneously collected using a water immersion objective. Emission signals were divided by a dichroic mirror (cut off = 630 nm) and projected onto the EMCCD camera (Andor). Data were recorded with 100 ms frame integration time and then processed by IDL script and analyzed by Matlab scripts.

### smFRET data analysis

To generate the FRET histogram, 21 frames of 20 short movies were collected at different imaging locations, yielding >6000 molecules. Alternating lasers (green and red) were used to excite sequentially both Cy3 and Cy5 (10 frames for Cy3, 1 frame dark and 10 frames for Cy5) to exclude the donor-only molecules from the histogram at the low FRET region. Furthermore, the donor leakage was corrected based on FRET values of donor-only molecules. Origin 2018 was used to fit the Gaussian distributions with an unrestrained peak centre position of the individually corrected and normalized histogram. The restrained peak centre position was used for RecA bound histogram. All the results and standard deviations shown in histogram fittings were calculated by incorporating more than three independent experiments.

### smFRET real time experiment

The smFRET real-time G4 unwinding assays using UvrD, Rep and Rep-X were carried out with a flow chamber and the same micro-fluidic imaging channel described above. A small piece of the plastic reservoir was placed above the one hole at the one end of the chamber and corresponding other holes at the opposite end connected with a silicone tube with a syringe pump (Harvard Apparatus, MA, USA). Protein (100 nM) and ATP (2 mM) suspended in imaging buffer was loaded into the reservoir. The real-time FRET images were collected by passing solution through the imaging chamber that contained dual-labeled (Cy3 and Cy5) partial duplex DNA with G4 via a silicone tubing at a flow rate of 20 μL/s. The smFRET time trajectories were analyzed using Matlab scripts. Using the individual single-molecule real-time flow traces, the binding kinetics were calculated from moment of flow to the moment of first irreversible FRET decline, and the G4 unwinding kinetics were measured from the fluctuation in FRET signal. In all three helicase experiments, more than 150 molecules were quantified for all kinetic calculations. Additionally, the DNA construct annealed either in 50 mM NaCl and diluted at 100 mM KCl or directly 100 mM KCl resulted in the same unwinding activity of all proteins tested here.

### smFRET unwinding assays

FRET spots disappeared as the helicase uncoiled G4, allowing the duplex to unwind. To calculate the unwinding rate, short movies (2 s) were recorded just after the injection of the respective helicase with or without ATP in the imaging buffer. The short movies were continuously recorded at different imaging areas and continued for almost 10 min. The number of FRET spots (300–400 molecules per view) counted over time. Consecutive three counted spot numbers and also time were averaged and plotted using Origin. Using the single exponential decay fitting curve, the unwinding rate was determined. These unwinding experiments were performed with different ATP concentrations.

RecA (1 μM; from NEB) and 2 mM ATP were used for RecA assembly assays using the same imaging buffer as used in the helicase experiment. Short movies were collected at different imaging areas over time, and FRET histograms were generated. The kinetic rate of RecA filament formation was calculated from fitting histogram by Gaussian distribution at different times after RecA addition.

### G4 ligand dislodge assay

The ligand displacement assay was carried out by applying 10 μM ligand to the immobilized G4-containing sample chamber. Unbound ligands were washed away, and100 nM of respective helicase with 2 mM of ATP was flowed in the same imaging buffer. Short movies were recorded and the spots were counted over time as described above. To monitor the real-time ligand dislodging from G4, ligand was applied followed by washing out the free ligand and finally adding the respective helicase with ATP while recording one continuous long movie.

After washing the excess ligands, RecA (1 μM) supplemented with ATP (2 mM) was applied and short movies (∼2 s) were recorded at different time intervals to generate histograms for kinetic analysis. Subsequently, long movies were recorded for 2 min to observe the molecular behaviour.

## RESULTS

### Cells lacking *rep* or *recA* are sensitive to G4 stabilizing ligands

Unresolved G4 structures have been shown to disrupt DNA replication and repair with potentially lethal effects (12). Genome maintenance proteins are implicated in regulating the formation and unwinding of G4 structures *in vitro* and *in vivo* (2). However, it remains unclear which of these proteins are required for G4 tolerance. To explore the roles that genome maintenance proteins play in tolerating G4s, strains lacking selected set of proteins were grown in the presence of ligand stabilized-G4s. We tested the ability of mutant strains with deletions in *rep::kan, uvrD::kan, recA::kan, recQ::kan* to grow on LB plates containing two structurally distinct G4 stabilizers, N-methyl mesoporphyrin IX (NMM) or BRACO-19. To determine the G4 stabilizer concentrations for these experiments, the sensitivities of *E. coli* MG1655 and of an *imp-4213* strain were tested using a range of NMM and BRACO-19 concentrations. Minimal concentrations at which the strains had unaffected colony-forming units in spot plating experiments were 20 μM NMM for MG1655 and 75 μM BRACO-19 for the *imp-4213* strain.

**Figure 1.**
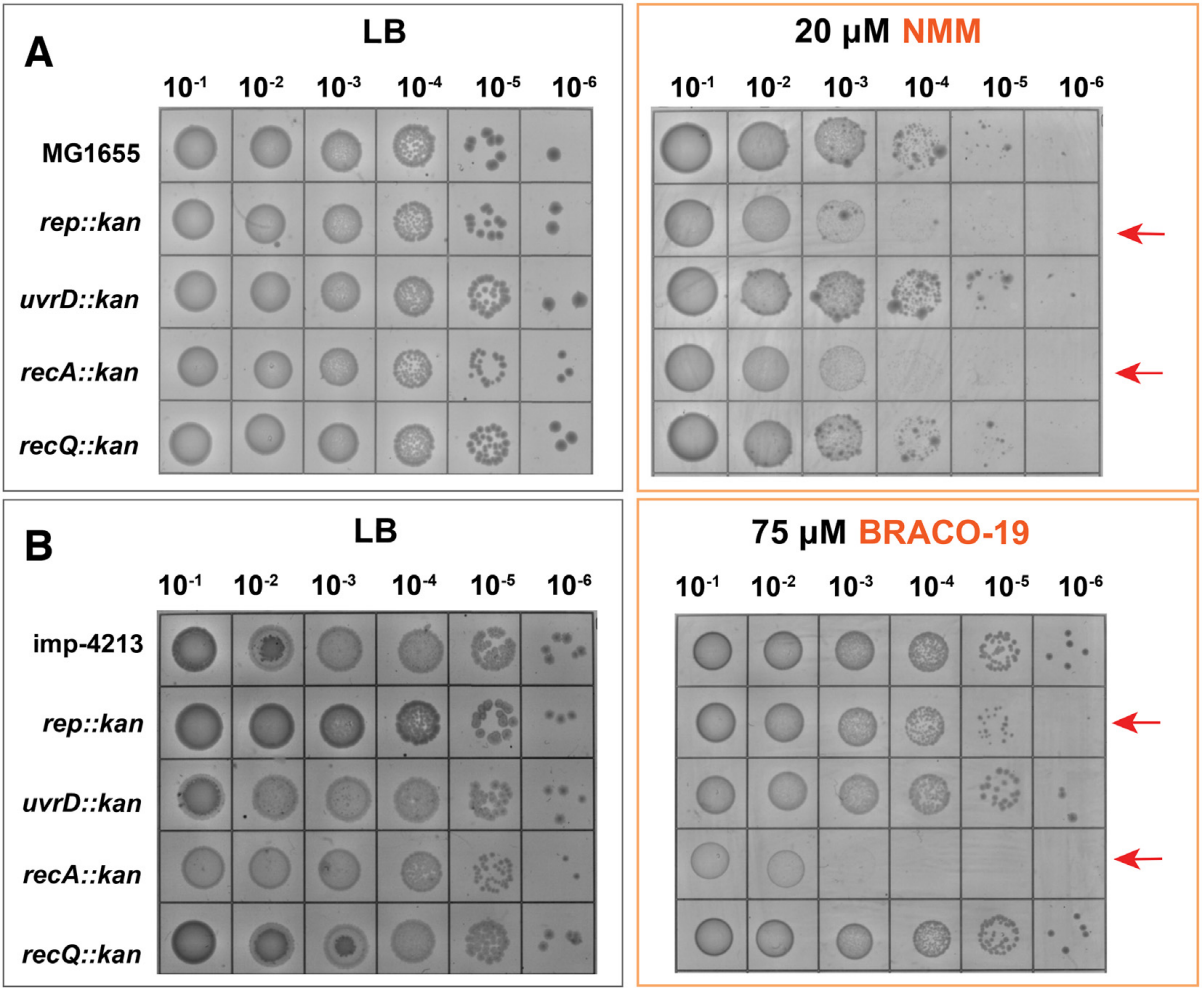
G4 ligand sensitivity assay. (**A**) Deletion strains, *rep::kan, uvrD::kan, recA::kan, recQ::kan* grown on LB (left) and with 30 μM NMM (right). (**B**) Deletion strains, *rep::kan, uvrD::kan, recA::kan, recQ::kan* grown on LB (left) and with 75 μM BRACO-19 (right) in imp-4213 background to increase cell permeability.

While the *uvrD* and *recQ* deletion strains tolerated the G4 ligand toxicity, strains lacking either *rep* or *recA* were sensitized to NMM (Figure 1A). None of the deletion strains tested were susceptible to BRACO-19. We hypothesized that BRACO-19 was unable to reach high enough intracellular concentration to have an effect. When the *rep* and *recA* deletions were transferred to an *imp-4213* background (known to have a hyperpermeable membrane) both strains were sensitized to BRACO-19 (45). We therefore used the *imp-4213* background for all BRACO-19 experiments.

Surprisingly, single gene knockout strains *uvrD::kan* and *recQ::kan*, which both encode known G4 helicases (23,31), did not decrease cell viability in the presence of NMM or BRACO-19 (Figure 1A, B). This indicates that these helicases may not be as important for the tolerance of unresolved G4 structures *in vivo*. Another group of genes, including *uvrA*, the G4-interacting *mutS* (46), and the G4 helicase *dinG* (47), were found to be important for NMM tolerance, but not BRACO-19 (Supplementary Figure S1).

### Rep-X and Rep unwind G-quadruplex proficiently

The G4-ligand sensitivity shown above strongly suggests that Rep, but not UvrD, is critical in overcoming G4 ligand-induced toxicity. This observation is puzzling in two aspects. First, the stark contrast between Rep and UvrD is not expected because they share structural and functional similarities as closely related members of Superfamily I helicase (48,49). Second, Rep has not been shown to unfold G4 whereas G4 DNA unwinding has been demonstrated for *E. coli* UvrD (31). Nevertheless, based on the *in vivo* G4 ligand sensitivity results, we hypothesized that Rep may be capable of unwinding G4 better than UvrD.

**Figure 2.**
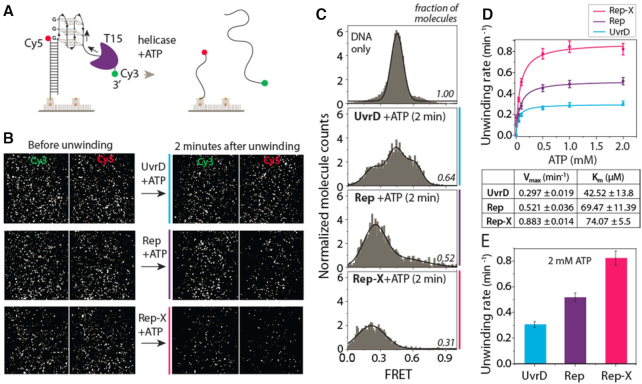
Rep-X and Rep unwind G4 more proficiently than UvrD. (**A**) Schematic diagram of DNA construct with G4 and T15 tail at the 3′ end and expected loss of Cy3 strand upon completion of unwinding. (**B**) Representative field of view before and after unwinding. Each white dot represents fluorescent DNA molecules. The FRET efficiency and the number of molecules decreases over time of unwinding for all three proteins (100 nM) added with ATP (2 mM). (**C**) FRET histograms taken before unwinding (top, DNA only) and after 2 min of unwinding for all three cases. (**D**) Unwinding rate calculated by the molecule count over time and the *V*_max_ and *K*_m_ deduced from Michaelis–Menten fit. (**E**) The unwinding rate of three proteins at 2 mM ATP condition.

To test this hypothesis, we compared G4 unwinding by Rep, UvrD and Rep-X, a Rep variant that displays heightened duplex DNA unwinding activity (34). To investigate the G4 resolving activity of three different helicases by single-molecule (sm) FRET, we prepared a substrate containing a duplex DNA (18 bp) and ssDNA composed of a G4 (four repeat of TTAGGG which folds into G4) and a T15 tail for helicase loading. The donor (Cy3) and acceptor (Cy5) dyes were situated across G4-T15 to probe the helicase binding to the ssDNA tail followed by G4 and duplex (18 bp) unwinding (Figure 2A). The complete unwinding of the duplex leads to disappearance of Cy3 signals and concomitant loss of FRET. Representative fields of view recorded before and after the addition of individual helicases (100 nM) and ATP (2 mM) show reduction in number of both Cy3 and Cy5 molecules over time in all three cases (Figure 2B).

**Figure 3.**
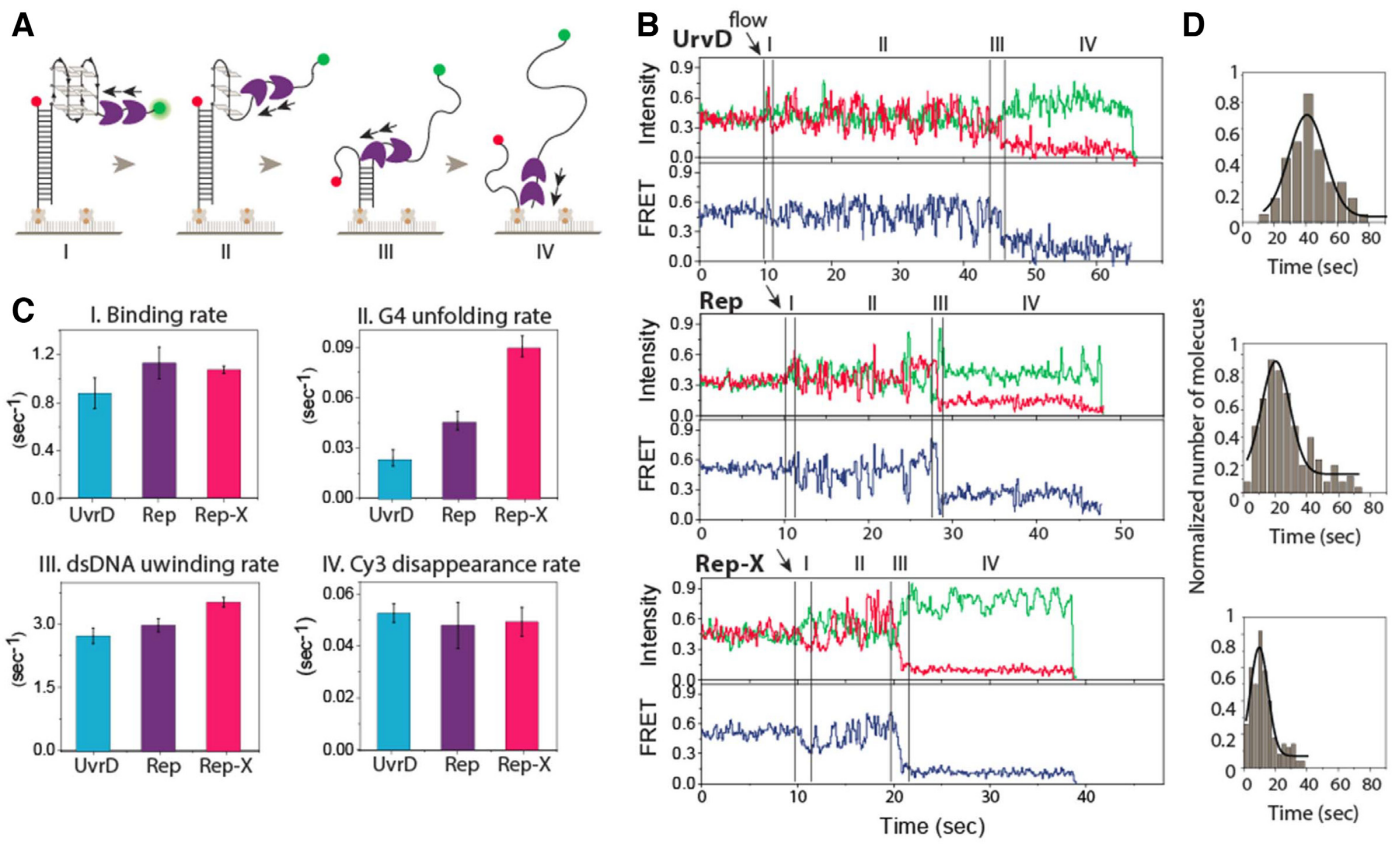
G4 unwinding mechanism involves multiple unfolding and refolding cycles. (**A**) Schematic diagram of four stages of unwinding activity: (I) binding, (II) G4 unfolding, (III) duplex unwinding and (IV) Cy3 departure. (**B**) The representative real-time smFRET unwinding traces of UvrD, Rep and Rep-X (100 nM protein and 2 mM ATP) and four stages as stated in (A). (**C**) Rates of I, II, III and IV calculated from smFRET traces plotted with SEM (>150 traces per condition). (**D**) Gaussian fit of the dwell time histogram of G4 unwinding for the corresponding proteins.

FRET histograms were built by collecting FRET values from >4000 molecules obtained from 20 different fields of view. The G4 DNA exhibits the FRET peak at ∼0.5 due to the distance between Cy3 and Cy5 separated by the G4 and T15 tail (Figure 2C). After addition of UvrD, Rep, or Rep-X with ATP, the total number of molecules decreased over time, as expected from G4 unwinding. Approximately 75%, 50% and 25% molecules disappeared in two minutes for Rep-X, Rep and UvrD, respectively (Figure 2B, C). In addition, the FRET histogram peaks shifted to lower FRET ∼0.2, especially for Rep and Rep-X, signifying faster unfolding of G4. The widely distributed histogram with additional peak ∼0.6 for UvrD arises from slower G4 unwinding, which is evidenced by the extended period of repetitive FRET fluctuation (see Figure 3B). To test the ATP dependence in unwinding, we titrated ATP concentration from 1 μM to 2 mM while keeping the same protein concentration (100 nM). For kinetic analysis, we counted the number of Cy3 molecules by capturing short movies (∼2 s) sequentially at different fields of view to avoid photobleaching (Supplementary Figure S2). The unwinding rates from three proteins were then fitted to the Michaelis-Menten plot from which we obtained *V*_max_ and *K*_m_ for Rep-X, Rep and UvrD (Figure 2D). The unwinding rate at 2 mM ATP and *V*_max_ for Rep and Rep-X was two and three times higher than UvrD, respectively (Figure 2E). Taken together, these data indicate that Rep-X and Rep unwind G4 more proficiently than UvrD.

To measure the extent to which G4 acts as a barrier in unwinding, we performed the same Rep induced unwinding assay using T15, T40 tail without G4 and G4-duplex with T15 tail (Supplementary Figure S3). T15 is the same tail length used in the G4 construct used above whereas T40 is the length sum of G4 and T15 (24+15 = 39), keeping the same 18bp duplex. G4-duplex contain the same length and identical nucleotides composition as of (TTAGGG)_4_-T15 construct that use here for unwinding experiment. This control can directly reflects the G4 versus G4-duplex unwinding. Surprisingly, the *V*_max_ for T15 and G4-duplex are almost similar (∼1.37 ± 0.03/min and ∼1.32 ± 0.05/min) and less from T40 (∼2.86 ± 0.03/min). Those rates reflect ∼2.5–5 times faster unwinding than that of G4 containing unwinding, clearly indicating a delay due to G4 unfolding. The *K*_m_ of T15 and G4-duplex remains comparable to the G4-containing substrate, but ∼4 times less for T40 (Supplementary Figure S3). All the unwinding experiments were carried out by applying protein and ATP together. Much slower unwinding was observed when protein was loaded before the ATP addition, indicating a requirement of multiple or successive protein loading for efficient unwinding (Supplementary Figure S4).

### Repetitive G4 unfolding is the rate limiting step

To gain mechanistic insight into the G4 resolving activity of three helicases, we examined the smFRET traces taken in real-time flow measurements in which data were acquired while the protein and ATP were added to the G4-containing DNA substrate. This experiment provides insights into all stages of the protein activity in one contiguous movie (25,50,51). The four stages include (I) helicase loading, (II) G4 unfolding, (III) duplex unwinding and (IV) completion of unwinding (Figure 3A). The representative smFRET traces of UvrD, Rep and Rep-X display all four stages of activity (Figure 3B). The flow of protein (100 nM) and ATP (2 mM) started at 10 s (Figure 3B, arrows) in all cases. Immediately after flow, we observed a spike in Cy3 signal as expected from the protein loading at the 3′ end, which exhibits protein induced fluorescence enhancement (PIFE) (Figure 3B, I) (52,53). The binding rate calculated from over 150 traces is similar for all three proteins (Figure 3C, I). Helicase binding is followed by a long period of FRET fluctuations, which is similar to the repetitive G4 resolving activity seen in other G4 helicases such as RHAU, BLM, and WRN (Figure 3B, II) (19,20,25). We interpret this pattern as emerging from repetitive cycles of unwinding and refolding of G4 which acts as a physical barrier. Interestingly, such G4 unwinding takes 35–45 s for UvrD, but only 10 and 20 seconds for Rep-X and Rep, respectively (Figure 3D). Therefore, unlike the binding rate, the G4 unwinding rate shows a significant difference between the helicases with the rank order of Rep-X > Rep > UvrD (Figure 3C, II).

The completion of G4 unwinding is followed by the duplex unwinding, which is denoted by a decrease in FRET (Figure 3B, III). Compared to the G4 unwinding, the rate of duplex unwinding is up to two orders of magnitude faster. Interestingly, the duplex unwinding rate was similar for all three proteins, in the range of approximately 3 s^−1^ (Figure 3C, III). The last stage is the ejection of the Cy3 strand, which is not part of G4 or duplex unwinding (Figure 3B, IV). The delay of Cy3 strand departure can be due to the helicase holding onto the tracking strand before releasing it into solution (more traces in Supplementary Figure S5A). The repetitive fluctuation was not present but delay of Cy3 strand departure observed when the same helicase activity was probed on T15 partial duplex (lack of G4) (Supplementary Figure S5B). Taken together, we demonstrate for the first time that Rep-X, Rep and UvrD are capable of unfolding G4 powered by ATP hydrolysis, albeit at different rates. Interestingly, the G4 unwinding mechanism of three proteins follow the similar pattern of repetitive unfolding and refolding cycles.

### Role of tail length and G4 conformation.

To further probe the unwinding abilities of the three helicases, we tested the G4 substrate with a shorter 3′ ssDNA tail, T9 (Supplementary Figure S6A). In all three cases, only 40% of unwinding (loss of Cy3 molecule) was observed in 10 minutes (Supplementary Figure S6B). The rest 60% of molecules remained protein bound, indicated by the overall FRET shift represented in FRET histogram. The kinetics analysis showed that the rate of unwinding is almost equal of three helicases (0.15671 ± 0.02581 min^−1^ for UvrD, 0.16269 ± 0.02472 min^−1^ for Rep and 0.18364 ± 0.019 min^−1^ for Rep-X), which are substantially lower than that of T15 tailed substrate (Supplementary Figure S6C). Such difference between T9 and T15 might be due to the tail length required for proper loading and G4 unfolding which involves iterative cycles of unfolding and refolding.

So far, we used non-parallel G4 as unwinding substrates. Next, we tested the parallel G4 unwinding by preparing a DNA construct bearing c-Myc sequence with T15 tail at the 3′ end (Supplementary Figure S7A). Parallel G4s are generally more stable than antiparallel G4s, making them more challenging to unwind. Upon addition of individual helicase (100 nM with 2 mM ATP) only about ∼10% Cy3 signal for both UvrD and Rep disappeared whereas ∼75% for Rep-X was lost in 10 minutes (Supplementary Figure S7B), signifying the efficient unwinding activity of Rep-X for parallel G4. The negligible and much slower unwinding rate indicates that UvrD and Rep are not capable of resolving the parallel G4 (Supplementary Figure S7C). The *V*_max_ is ∼0.44 min^−1^ with *K*_m_ is ∼215 μM from Michaelis–Menten fitting is approximately two times lower *V*_max_ and three times higher *K*_m_ than the non-parallel G4 shown previously (Supplementary Figure S7D, E). Interestingly, Rep-X doesn’t show unwinding of parallel c-Myc G4 with T9 tail (Supplementary Figure S7F), although T9 is sufficient for Rep-X to unwind non-parallel G4. This likely reflecting that the cross-linked conformation of Rep-X may require longer tail for efficient unwinding of a tightly folded parallel G4.

### Rep and Rep-X dislodge G4 ligand and unfold G4

**Figure 4.**
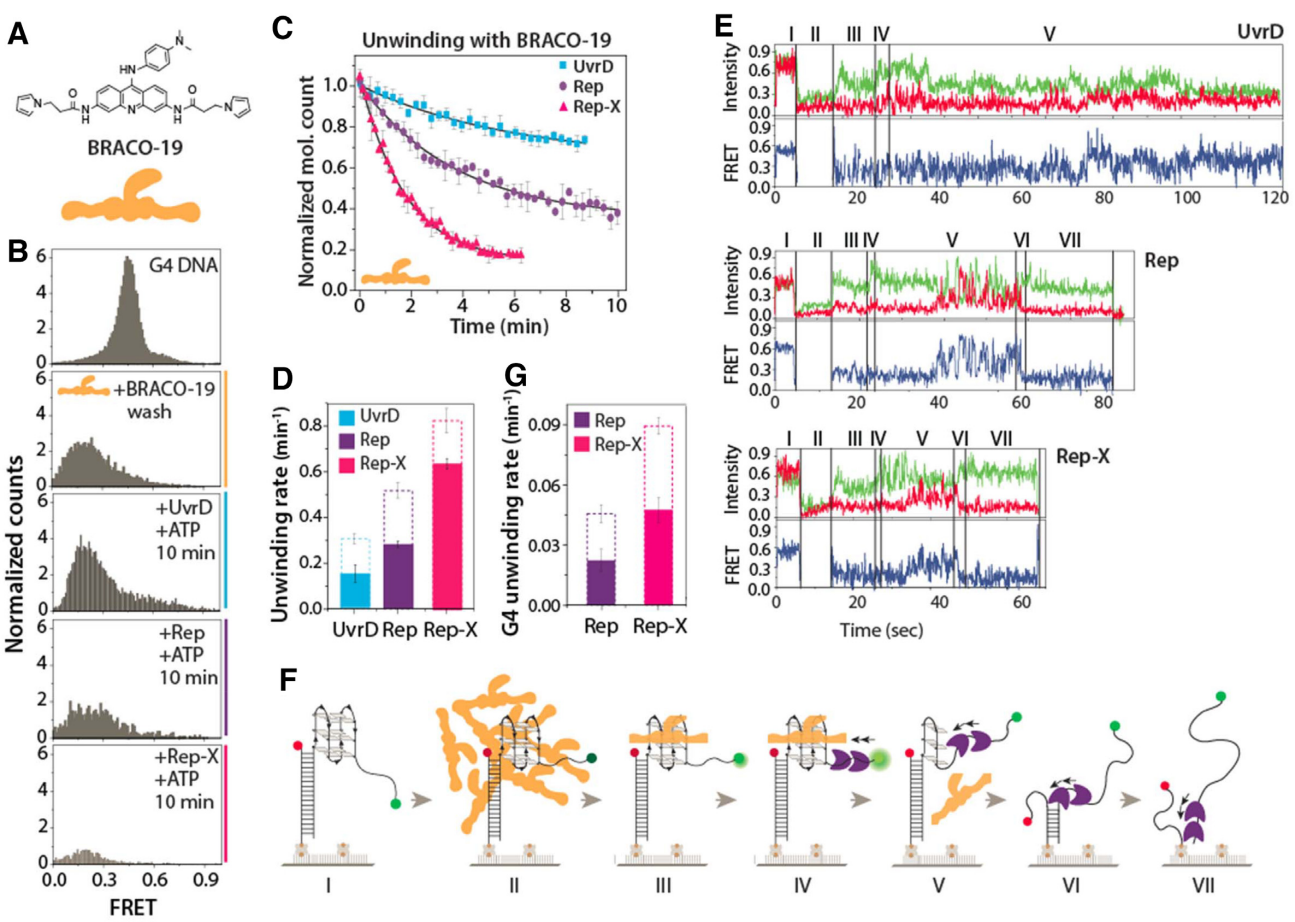
Rep and Rep-X dislodge G4 bound ligand. (**A**) Chemical structure of BRACO-19 and a pictorial depiction of BRACO-19 in orange. (**B**) Normalized smFRET histogram of G4 DNA (top), with BRACO-19 after buffer wash of excess ligand (second) and then helicases with ATP taken after 10 minutes (bottom three). (**C**) Single exponential fitting of unwinding kinetics for UvrD, Rep and Rep-X in ligand-bound condition. (**D**) Bar graph of the overall unwinding rate (Cy3 departure rate) in presence (filled) and absence (blank with dotted line) of G4-ligand. (**E**) Representative smFRET flow traces with demarcations for sequential steps including BRACO-19 addition, buffer wash, protein and ATP addition, G4 resolution, duplex unwinding and Cy3 strand departure with each step marked I to VII. (**F**) Schematic diagram of G4 DNA (I), with excess ligand (II), after buffer wash of free ligand (III), helicase added (IV), helicase dislodging ligand and resolving G4 (V), duplex unwinding (VI) and Cy3 departure (VII). (**G**) Rep and Rep-X induced G4 unwinding rate in presence (filled) and absence (blank, dotted line) of bound ligand.

We showed above that UvrD, Rep and Rep-X helicase activity is sufficient to unwind G4 DNA structures. Based on the G4 ligand sensitivity result (Figure 1), we next asked if the G4 unfolding activity can lead to dislodging of the G4 bound ligand. To test this, we chose BRACO-19, a potent G4 ligand which was also tested in the sensitivity assay (Figure 4A, BRACO-19 drawn in orange). The addition of BRACO-19 to G4 and washing out excess ligand shifted the FRET peak from 0.5 to 0.2 primarily due to quenching of both FRET dyes (Figure 4B). When helicase (100 nM) and ATP (2 mM) were added, 25%, 65% and 85% of molecules disappeared for UvrD, Rep and Rep-X respectively after 10 min, signifying the removal of BRACO-19 and subsequent resolving of G4 and completion of unwinding (Figure 4B). For kinetic analysis, we counted the number of Cy3 molecules over time after the addition of helicase and ATP in the G4 ligand-bound condition (Figure 4C). The unwinding rate obtained from the fitted decay curve is substantially lower than that of G4 free of ligand, albeit in the same order of Rep-X > Rep > UvrD (Figure 4D and Supplementary Table S2).

To capture the sequence of events from the ligand binding, displacement, G4 resolution and duplex unwinding, we performed real-time flow measurement in which ligand and helicase were added in succession. In the representative single molecule traces (Figure 4E), we define seven sequential steps (I- VII) depicting distinguishable stages of helicase activity (Figure 4F). First, the flow of BRACO-19 to G4 at ∼5 s induced an immediate quenching of Cy3 and Cy5 signals (step II, 5–15 s). Second, the buffer wash of free ligand enhanced the Cy3 signal (step III, at ∼15 s) likely due to dequenching of Cy3 by removal of excess BRACO-19. Third, the helicase loading to ssDNA tail makes Cy3 signal brighter, exhibiting PIFE effect (step IV, at ∼25 s) (52,53). Fourth and fifth, the helicase dislodges the G4 ligand and resolves the G4 structure represented by a lag period of low FRET state and subsequent FRET fluctuation respectively (step V). The subsequent stages of VI and VII represent duplex unwinding and departure of Cy3 strand, respectively as seen before. Interestingly, the repetitive unwinding and refolding cycles corresponding to the G4 resolving activity (step V) were observed for Rep and Rep-X but not for UvrD. The lack of G4 resolving activity by UvrD likely indicates that UvrD cannot remove BRACO-19 bound to G4 (more traces in Supplementary Figure S8). This result is in agreement with the G4 ligand sensitivity result which showed that deletion of *uvrD* had no effect whereas deletion of Rep reduced the tolerance for G4 ligand-mediated toxicity. However, we calculated the rate of protein binding, G4 unwinding and duplex unwinding for Rep and Rep-X. While rate of protein binding (also for UvrD) and duplex unwinding are almost similar to the rate measured for Rep and Rep-X in the absence of BRACO-19, there was a significant difference in the rate of G4 unwinding, strongly indicating the delay due to dislodging of BRACO-19. The G4 unwinding rate was approximately two-fold lower than that obtained without G4 ligand due to the extra time it took for the ligand removal prior to G4 unwinding (Figure 4G and Supplementary Table S2). Therefore, the data indicate that Rep and Rep-X are able to dislodge the G4 ligand proficiently whereas UvrD cannot.

### RecA recombinase resolves G4 and dislodges G4 ligand by filament formation

**Figure 5.**
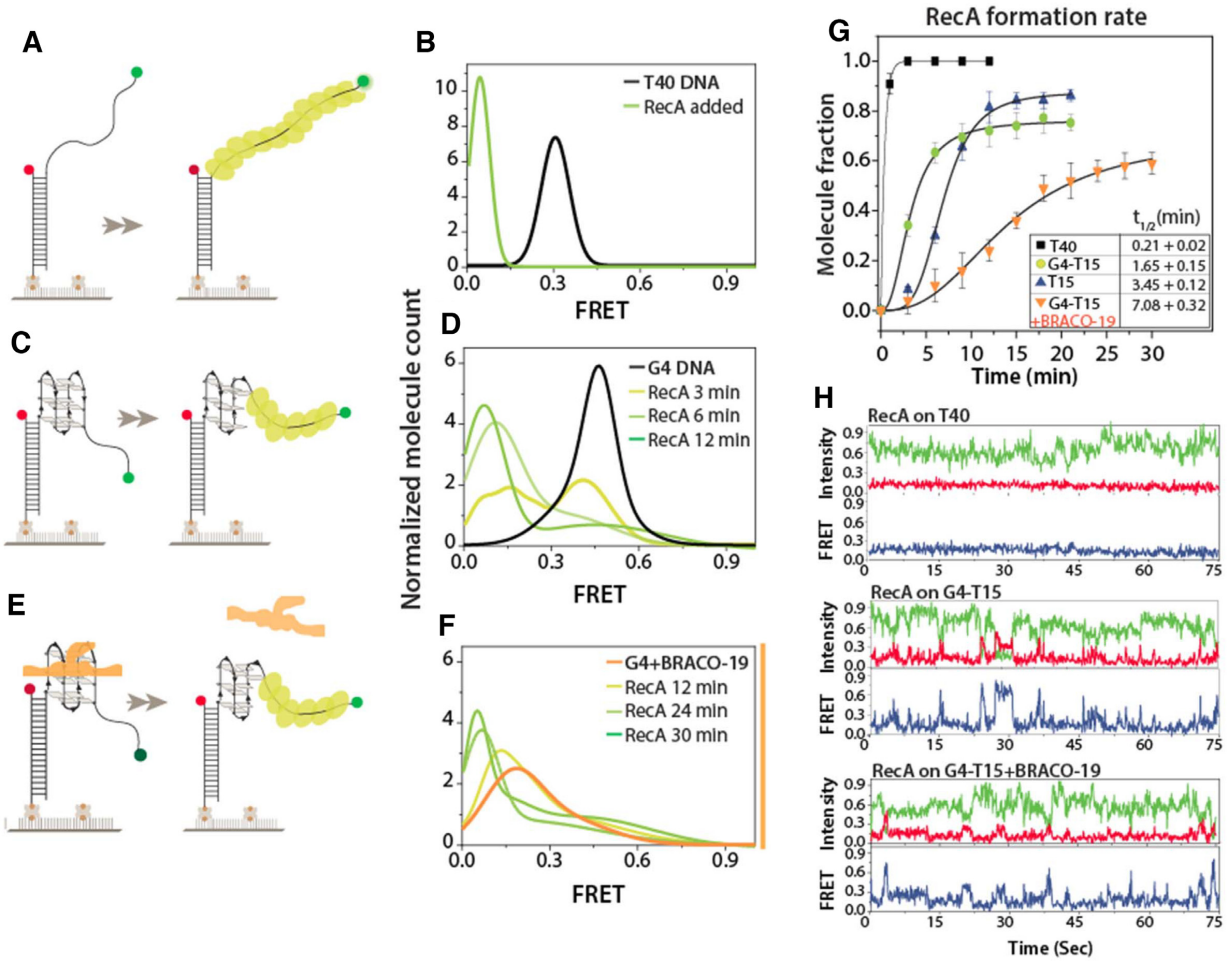
RecA assemble on G4 and dislodge ligand. (**A, C**, **E**) Schematic smFRET model of partial duplex with T40 tail, G4-T15 and ligand-bound G4 (left panel) and the corresponding RecA filament formation (right panel). (**B**, **D**, **F**) The smFRET histogram of DNA only and ligand-bound G4 and with RecA (1 μM with 2 mM ATP) filament formation of corresponding DNAs over time. (**G**) Rate of RecA filament formation on T40, G4-T15, T15 and G4-T15 in ligand bound condition. (**H**) After RecA filament formation, the representative smFRET traces of T40 (top), G4-T15 (middle) and after dislodging G4 ligand (bottom).

The hypersensitivity of the Δ*recA* strains to G4 ligands led us to examine its activity on G4 DNA as well. RecA is essential for mediating homologous recombination required for maintaining genomic integrity (54). RecA binds ssDNA and forms a helical filament that becomes stable when at least six RecA monomers bind ssDNA of 18 nucleotides or longer (55,56). To establish the working condition, we used partial duplex FRET construct with a 3′-T40 tail to test RecA filament formation (Figure 5A). Addition of 1 μM RecA with 2 mM ATP immediately shifted the FRET peak from 0.3 (T40 ssDNA) to ∼0.05, which is consistent with stretching of the ssDNA due to stable RecA filament formation (Figure 5B). Next, we asked whether RecA can resolve a G4 structure through such filament formation. To test this, we applied RecA (1 μM with 2 mM ATP) to the non-parallel G4 with T15 tail (Figure 5C). Consistent with RecA-mediated G4 unwinding, the FRET peak shifted gradually from ∼0.5 to ∼0.05, with ∼75% molecules shifting within 12 minutes (Figure 5D). By contrast, when RecA is added to G4 DNA without tail, the FRET peak remained unchanged, consistent with a requirement for a ssDNA tail for RecA loading (data not shown). As a control, RecA was added to a T15 DNA without a G4, and ∼80% molecules showed FRET shift from ∼0.75 to ∼0.2 after 12 minutes (Supplementary Figure S9A, B). Comparing the RecA bound FRET peak of T15 at ∼0.2 with G4-T15 at ∼0.05 suggest that RecA disrupt the G4 structure as almost same length T40 show RecA bound FRET at ∼0.05. The similar RecA experiment performed on parallel c-Myc-T15 construct reveals that RecA cannot disrupt the tightly folded parallel G4 structure (Supplementary Figure S9E, F). Therefore, the RecA filaments can resolve non-parallel G4 provided that a ssDNA tail is present.

Next, we sought to test whether RecA can dislodge BRACO-19 and resolve ligand-bound G4 DNA (Figure 5E). Upon the addition of RecA (1 μM with 2 mM ATP), the FRET peak gradually shifted to ∼0.05, with ∼60% of the population shifting in 30 min (Figure 5F).

Considering half-time (*t*_1/2_) i.e. 50% of RecA assembled on the respective construct, we compared the rate of RecA formation. The highest rate of RecA formation was obtained for T40, followed by G4-T15, T15, and the ligand bound G4-T15 (Figure 5G). We noticed that the fraction of RecA formation reached 100% for T40, but plateaued at 70–80% for G4-T15 and T15 and 60% for the BRACO-19 bound G4-T15. The single molecule traces taken after filament formation revealed a stable low FRET for T40, suggesting that T40 allows stable filament formation. By contrast, the traces taken for G4-T15 displayed dynamic FRET fluctuations, likely representing binding and dissociation of RecA on the G4-T15 substrate (Figure 5H). The similar pattern of FRET fluctuation was observed for T15, in agreement with 18 nt required for a stable nucleation (more traces in Supplementary Figure S10) (56–58). Hence, the FRET fluctuation observed for G4-T15 likely indicates partial unfolding and refolding of G4 in dynamic exchange. This pattern indicates that RecA filament cannot unfold G4 completely in this condition. If the filament completely unfolded G4, smFRET traces would show a stable low FRET as seen in the case of T40 since the total length of G4-T15 is 39 nucleotides. Nevertheless, the dynamic unfolding and refolding from the G4 state mediated by RecA is sufficient to remove the G4 ligand. Interestingly, in the presence of sodium buffer, which is less stabilizing for G4 folding, we observed a stable low FRET on G4-T15, indicating complete unfolding induced by RecA (Supplementary Figure S9C, D). Therefore, we conclude that RecA dislodges the G4 ligand via forming filament on single stranded tail and invading into G4, which is consistent with our *in vivo* observation (Figure 1).

## DISCUSSION

### Rep is a newly identified G4 resolving helicase

It has been estimated that human genome contains over 350 000 potential G4 forming sequences (PQS) whereas *E. coli* genome has over 3000 (9). The PQS is distributed unevenly with high enrichment near sites of replication of origin, transcription, translation and telomerase maintenance, strongly suggesting that G4 plays important regulatory roles (5–10). Nevertheless, unresolved G4 structures have been shown to cause genome instability (2). Therefore, cells have evolved specialized helicases to recognize and unwind G4 structures and thereby prevent genomic instability (31). *In vitro*, bacterial RecQ helicases can unwind G4 DNA and an X-ray crystal structure identified the presence of a guanine-specific binding pocket on the surface of the helicase that can sequester guanine bases from unwound G4 DNA (23). However, it remains unclear which helicases are responsible for G4 tolerance in cells. Here, we report an unexpected role of Rep, but not RecQ or UvrD, in tolerating G4 ligand toxicity in cells. Strikingly, we show that Rep is a robust G4 resolvase whereas UvrD has much weaker activity. We also show that Rep's strong G4 unwinding activity dislodges G4 ligand whereas UvrD’s weaker G4 unfolding is insufficient to remove G4 ligands. Rep and UvrD are both 3′ to 5′ helicases that share 40% homology (27) and have been hypothesized to be redundant in function due to the lethality caused by *rep, uvrD* double mutants (59). Later studies, however showed that UvrD is functionally distinct from Rep (49,60). The difference between Rep and UvrD in G4 unwinding may be due to the different binding preferences i.e Rep has high affinity to 3′ tailed ssDNA whereas UvrD prefers associating at junctions between ssDNA and duplexes (61). Efficient loading of Rep at 3′ tails may facilitate its translocation on ssDNA, powering it forward for G4 resolution.

### Repetitive G4 unfolding is a shared mechanism

Interestingly, each of the helicases studies here shared a common mechanism of G4 in which repetitive cycles of G4 unwinding and refolding continued until the G4 was completely unfolded and the helicase made its way into the duplex DNA. Although the dye position in this substrate only indirectly reports on G4 unwinding, we chose the terminal position of Cy3 based on our previous study in which Cy3 at the entry of G4 i.e. junction between G4 and ssDNA tail prevented G4 helicases from properly loading on the substrate (25). This position of Cy3 provides an advantage of measuring both loading of the protein and subsequent G4 unfolding activity in repetitive manner. Such repetitive fluctuation was not present when the same helicase activity was probed on a substrate which is devoid of G4 structure (Supplementary Figure S5B). However, the repetitive FRET fluctuations we observe for all three helicases here are reminiscent of the unwinding mechanism observed for an unrelated G4 resolvase, DHX36. While the DNA-G4 unfolding by DHX36 was somewhat similar to the pattern seen here, it never resulted in subsequent duplex unwinding (25), likely due to a limited G4 disrupting activity (62). In addition, DHX36 exhibits distinct stepwise unfolding of RNA-G4 which occurs at ∼20-fold slower rate (25) than that acquired for Rep, Rep-X and UvrD. We interpret the FRET pattern shown in Figure 3B as repetitive and partial unfolding of G4 which eventually leads to complete G4 unfolding followed by duplex unwinding for all three helicases. Interestingly, the duration of the repetitive unfolding activity scaled with the unwinding strength: UvrD, the weakest G4 resolvase, spent more time in the repetitive cycles of unfolding motion than Rep or Rep-X. Such motion is also shared by many other G4 unwinding enzymes (22,25,62,63), suggesting that repetitive unfolding may be a conserved mechanism used by many helicases to overcome G4 barriers. The four- to five-fold slower unwinding rate observed with G4 compared to the duplex DNA reflects that G4 is a highly stable structure that represents a high energetic and physical barrier.

### Rep unwinding of G4 requires non-parallel G4 conformation and sufficient tail length

Our results point out several characteristics of Rep's G4 unfolding activity. First, Rep can only unwind a non-parallel G4 structure which is thermally less stable than the parallel G4. By contrast, Rep-X is able to unwind even the parallel G4, suggesting a stronger G4 resolving power of Rep-X. Second, all three helicases, Rep, Rep-X and UvrD require a ssDNA tail of sufficient length for unwinding activity because T15 led to efficient unwinding whereas T9 did not. Based on the footprint of Rep of approximately eight nucleotides (28), the requirement for T15 suggest that more than one helicase may be required for unwinding of G4. This is consistent with the DNA duplex unwinding by UvrD and Rep, which also require dimer loading on ssDNA (28). The T15 requirement seen for Rep-X may arise from the crosslinked conformation of Rep-X that requires longer tail even for a monomer loading for unwinding. In addition, a longer ssDNA may be required to provide a docking site for retaining the helicase as it moves back and forth in order to effectively resolve the bulky G4 structure during repetitive cycles of G4 unwinding and reformation. This was evidenced in the case of T9 where all the helicases remained bound to the tail without being able to resolve the G4 (Supplementary Figure S6). By contrast, DHX36, a G4 resolvase specific for parallel G4 unwinding only required 9 bases of ssDNA for G4 resolving, displaying the same type of repetitive FRET fluctuations as a monomer (25,62). The DHX36 structure revealed an architecture for the protein that is highly specialized binding to parallel G4, making an intimate and extensive contact with all sides of the parallel G4 structure (62). Due to this strong interaction, DHX36 may not require a long tail for resolving G4.

### RecA mediates G4 resolution and G4 ligand removal

It is interesting that RecA, a non-helicase protein, unfolded G4 DNA. RecA is a well-known recombinase in *E. coli*. In homologous recombination double-stranded DNA break repair, the broken DNA ends undergo resection in which RecBCD creates the 3′-terminated ssDNA onto which RecA is loaded, forming a filament on the DNA that executes a homology search to form a D-loop (64). In light of such function of RecA in homologous recombination, our results demonstrate that RecA filamentation promotes unwinding of G4 structures that might emerge within the resected ssDNA. RecA activity was also dependent on the length of ssDNA. Intriguingly, RecA could eject BRACO-19 and resolve G4s even on short DNAs that do not support RecA filament formation. Together, our result reveals that RecA as a recombinase is capable of resolving G4 structure on ssDNAs which can facilitate nucleoprotein filament formation needed for the homology search. This may be important for RecA activity in cells grown in the presence of G4-stabilizing ligands, as observed in experiments with Δ*recA* cells (Figure 1 and Supplementary Figure S1). Hypersensitivity of Δ*recA* cells to the G4 ligands may also be due RecA’s essential roles in repair of double-strand DNA breaks induced by the G4 ligands.

### Dislodging of G4 ligand by Rep and Rep-X

G4-interacting small molecules enhance G4 stability, thereby inhibiting cellular pathways including telomerase activity (20,65). G4 ligand binding to G4 structure can also downregulate gene expression (65). We demonstrate that the G4-bound ligands can be efficiently dislodged by Rep and RepX but not by UvrD. This finding is correlated with our *in vivo* observations in which the *Δrep* cells were sensitized to both NMM and BRACO-19 (Figure 1). Surprisingly, *ΔrecQ* and *ΔuvrD* did not show a decrease in cell viability in the presence of G4 ligands, indicating these helicases may not be important for the G4 tolerance. Although RecQ was previously shown to unwind G4 by a similar repetitive unfolding mechanism, the G4 unwinding rate of RecQ was 2–3 fold slower than that of Rep and Rep-X (23). In addition, RecQ mediated unwinding of G4 was inhibited by G4 ligand, an NMM derivative (18). Both findings are consistent with our *in vivo* result presented here, i.e. RecQ’s ability to unwind G4 and dislodge G4 ligand is weaker than that of Rep. Consistently, previous biochemical study showed that UvrD can unwind inter- and intra-molecular G4 structures, but the unwinding activity was impeded by G4 ligand binding (31). *In vivo*, Rep is known to function to rescue stalled replication fork. Previously, we demonstrated that the repetitive translocation activity of Rep on ssDNA occurs in such context i.e. on the lagging strand downstream of Okazaki fragment (66). We envision that the same may be true in the context of G4 bearing DNA, i.e. the repetitive cycles of translocation of Rep is useful to resolve the blockade of thermally stable G4 structure, thus removing the physical barrier. Together, our results suggest that Rep may be specialized in G4 removal activity, which may stem from its inherent ability to repetitively shuttle on ssDNA (66).

If both Rep and RecA are capable of resolving G4 structure and removing G4 ligand, why did *Δrep* and *ΔrecA* show sensitivity toward BRACO-19 and NMM? Rep and RecA could not compensate for the absence of each other (Figure 1). This may arise from the different pathways in which Rep and RecA participate. Rep functions to facilitate restarting of stalled replication forks (67) whereas RecA is responsible for forming a filament on single strand DNA during homologous recombination (68).

### Compare the activity of helicases and RecA in this study

The smFRET experiment does not allow us to measure the level of tension on single-strand DNA generated by the two proteins, our result reveals that helicases are more efficient at resolving G4 structure and removing G4 bound ligand in the context of the DNA constructs we tested here. The G4 unfolding rate by helicases is more than two orders of magnitude higher than RecA (Figures 4D vs. 5G). Likewise, the G4 ligand removal rate is approximately 5–7 fold higher in helicases than in RecA (Figures 4D versus 5G). In terms of RecA activity, longer filament of RecA formed on a longer ssDNA tail can lead to more robust G4 and ligand removal activity by generating higher tension.

### G4 unwinding activity contributes to genome integrity

**Figure 6.**
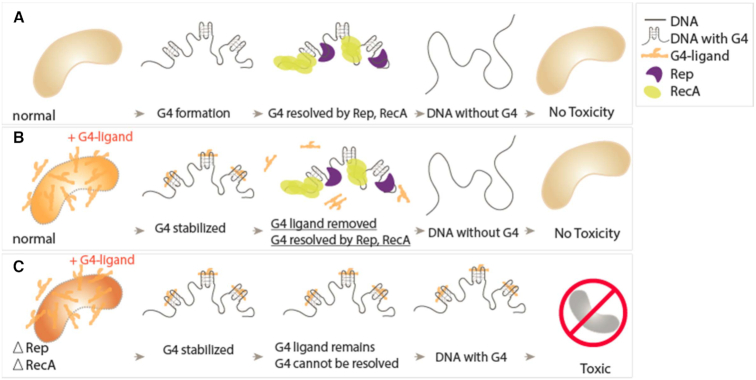
Model summary of possible causes of nontoxic and toxic cell. (**A**) Schematic of a normal cell containing G4-forming sequences. The G4 is resolved by either Rep or RecA. (**B**) The G4 ligand stabilized the G4 structure, and the G4 ligand can be dislodged by Rep or RecA. (**C**) When Rep or RecA are deleted, the cell is unable to dislodge the G4 ligand or resolve the G4 structure, which may lead to cellular toxicity.

Our study has led to identification of two key G4 resolvases, Rep and RecA that are critical for tolerance of ligand-stabilized G4 structures in *E. coli*. They are both important for overcoming cellular toxicity that arises from stably folded G4, such as G4s bound to G4 ligands (Figure 6). Despite their functional differences, both Rep and RecA are fueled by ATP hydrolysis and operate on ssDNA in a directional manner. G-rich sequences in ssDNA can easily fold into a thermally stable G4 structures, presenting blockades for proteins such as polymerases, helicases, and ssDNA-binding proteins of many types. In particular, the ssDNA targeted by Rep and RecA are high-risk spots that can lead genomic instability. For example, if G4 forms in ssDNA within a stalled replication fork, reversal or recovery of the stalled fork structure will be impeded. Therefore, Rep's ability to resolve G4 in this important junction is critical for rescuing replication forks and thereby preserving genome integrity. Next, if G4 formed in resected DNA cannot be removed, homologous recombination would fail, resulting in the prolonged double strand break without proper repair, which increases the risk of cell death. Therefore, the G4 resolving power of RecA is essential for ironing out the G4 structure and enabling RecA recombinase activity.

## Supplementary Material

gkaa442_Supplemental_File
