# Supplementary material for: E. coli Rep helicase and RecA recombinase unwind G4 DNA and are important for resistance to G4-stabilizing ligands
Source: Nucleic Acids Res. 2020 May 25;48(12):6640–53. doi: 10.1093/nar/gkaa442 (PMC7337899; doi:10.1093/nar/gkaa442)
Supplement: gkaa442_Supplemental_File [file gkaa442_supplemental_file.pdf]

SUPPLEMENTARY INFORMATION

E. coli Rep helicase and RecA recombinase unwind G4 DNA and are important for resistance to G4-stabilizing ligands

Tapas Paul[1], Andrew F. Voter[2], Rachel R. Cueny[2], Momčilo Gavrilov[1], Taekjip Ha[1,3,4], James L. Keck[2] and Sua Myong[1,3,*]

[1] Department of Biophysics, Johns Hopkins University, Baltimore, MD 21218, USA
[2] Department of Biomolecular Chemistry, University of Wisconsin School of Medicine and Public Health, Madison, WI 53706, USA
[3] Physics Frontier Center (Center for Physics of Living Cells), University of Illinois, 1110 W. Green St., Urbana, IL, 61801, USA
[4] Howard Hughes Medical Institute, Johns Hopkins University, USA.

* To whom correspondence maybe addressed. Tel: 410-516-5122; Fax: 410-516-4118; Email: smyong@jhu.edu

Supplementary information includes:
Supplementary Table 1
Supplementary Table 2
Supplementary Figure 1
Supplementary Figure 2
Supplementary Figure 3
Supplementary Figure 4
Supplementary Figure 5
Supplementary Figure 6
Supplementary Figure 7
Supplementary Figure 8
Supplementary Figure 9
Supplementary Figure 10

Supplementary Table 1. DNA oligonucleotides used in this experiment. All are 5' to 3' in direction.

TTAG4-T15/3'Cy3/	TGG CGA CGG CAG CGA GGC TT GGG TTA GGG TTA GGG TTA GGG TTT TTT TTT TTT TTT/3'Cy3/
c-Myc-T15/3'Cy3/	TGG CGA CGG CAG CGA GGC TT GGG T GGG TA GGG T GGG TTT TTT TTT TTT TTT/3'Cy3/
TTAG4-T9/3'amino C7/	TGG CGA CGG CAG CGA GGC TT GGG TTA GGG TTA GGG TTA GGG TTT TTT TTT/3'amino C7/
c-Myc-T9/3'amino C7/	TGG CGA CGG CAG CGA GGC TT GGG T GGG TA GGG T GGG TTT TTT TTT/3'amino C7/
T15/3'Cy3/	TGG CGA CGG CAG CGA GGC TTT TTT TTT TTT TTT/3'Cy3/
T40/3'Cy3/	TGG CGA CGG CAG CGA GGC TTT TTT TTT TTT TTT TTT TTT TTT TTT TTT TTT TTT TTT T/3'Cy3/
/5'Cy5/18merBio/	/5′Cy5/GCC TCG CTG CCG TCG CCA/3′Bio/
/5'Cy5/42merBio/	/5′Cy5/CCC TAA CCC TAA CCC TAA CCC TAA GCC TCG CTG CCG TCG CCA/3′Bio/

Supplementary Table 2. Helicase induced unwinding rate in presence and absence of ligand.

Unwinding rate (min^{-1})

	UvrD	Rep	Rep-X
Without ligand	0.3064 ± 0.02154	0.51767 ± 0.03383	0.82271 ± 0.05372
With ligand	0.14876 ± 0.03836	0.28001 ± 0.01542	0.63836 ± 0.02068

G4 unwinding rate (sec^{-1})

	Rep	Rep-X
Without ligand	0.0456 ± 0.00462	0.08934 ± 0.00413
With ligand	0.0223 ± 0.00562	0.0476 ± 0.00615

SUPPLEMENTARY FIGURE 1

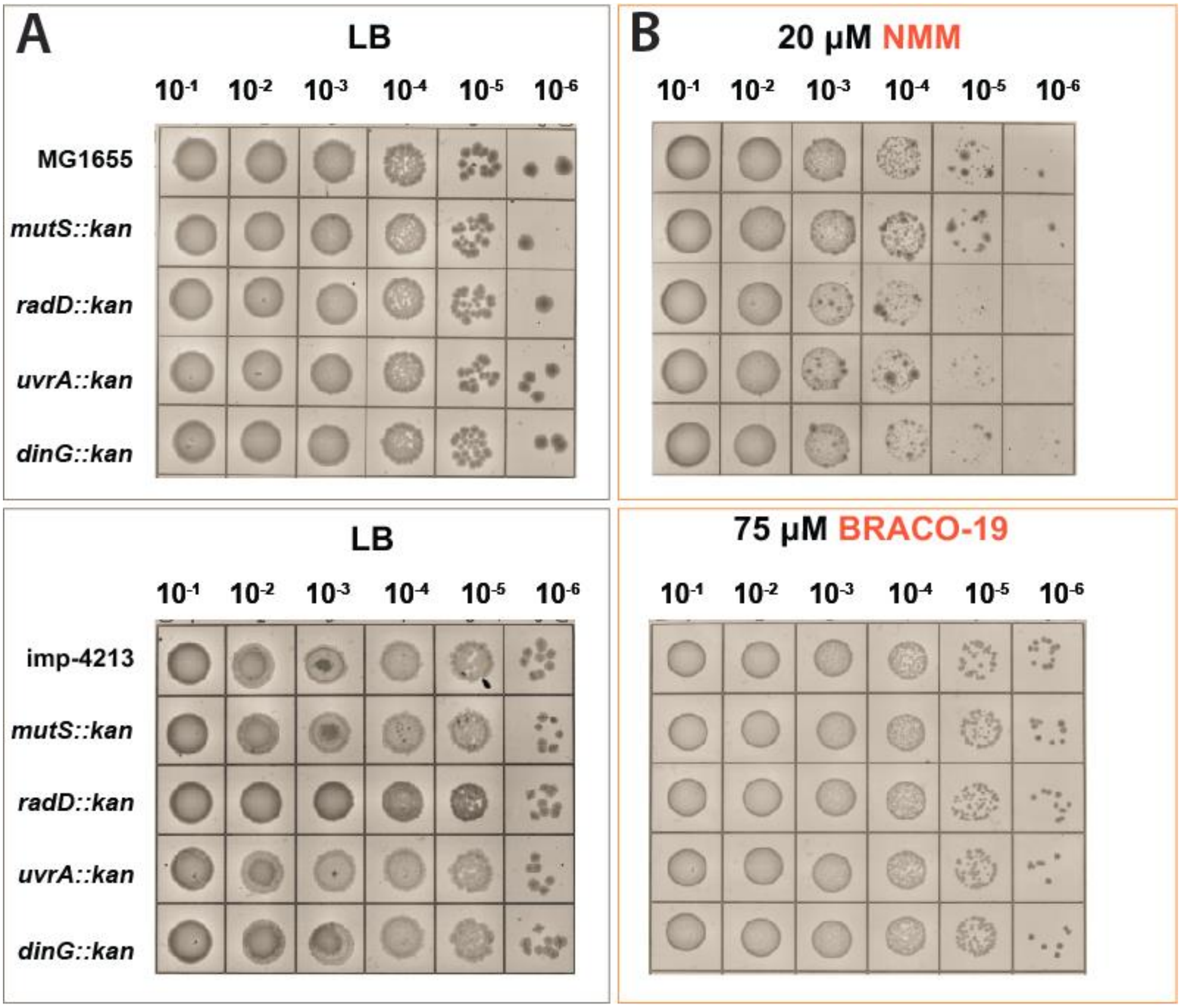

Figure S1. G4 ligand sensitivity assay. (**A, B**) Deletion strains, mutS::kan, radD::kan, uvrA::kan, dinG::kan grown on LB (left, top and bottom) and with 20 µM NMM (top, right) and with 75 µM BRACO-19 (bottom, right).

SUPPLEMENTARY FIGURE 2

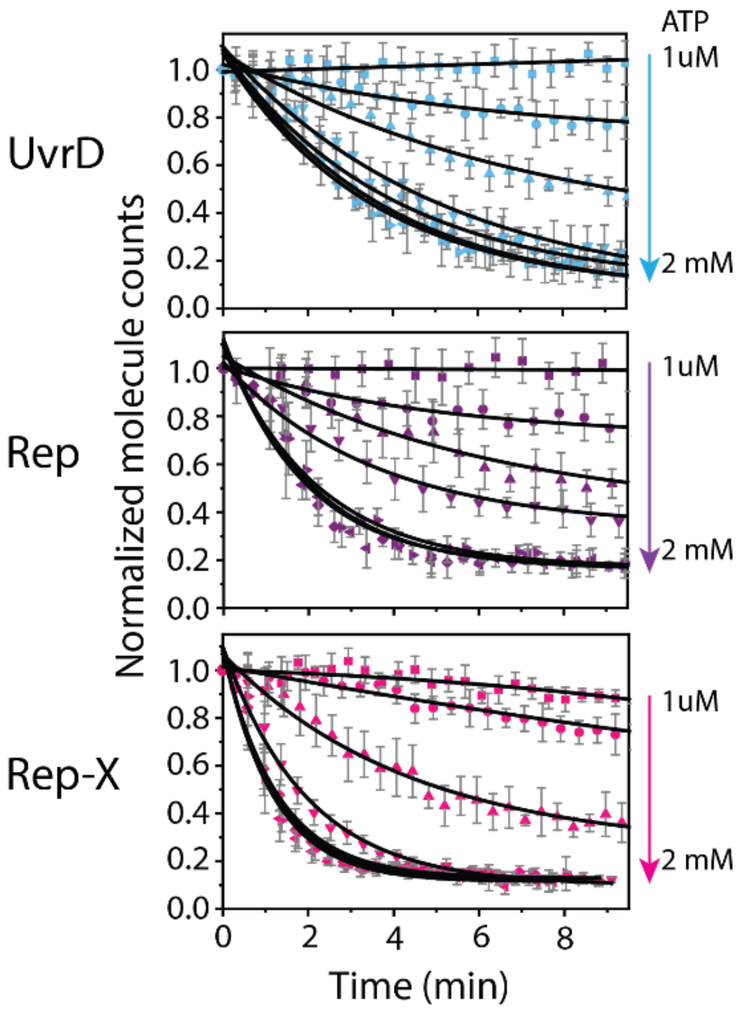

Figure S2. Unwinding of G4-15. Unwinding kinetics of G4-T15 using UvrD, Rep and Rep-X at different ATP concentration. The solid lines are the single exponential fitted decay.

SUPPLEMENTARY FIGURE 3

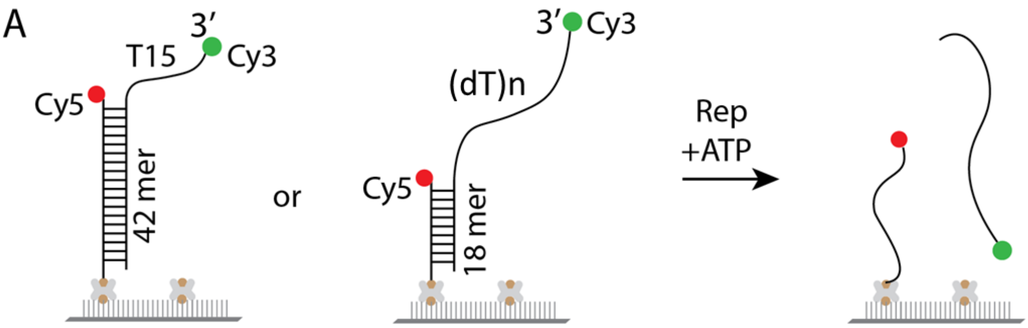

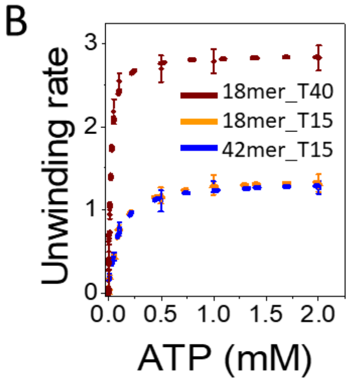

	V_{max} (min^{-1})	K_m (µM)
18mer_T40	2.86 ± 0.03	17.7 ± 2.3
18mer_T15	1.37 ± 0.03	74.6 ± 7.6
42mer_T15	1.32 ± 0.05	70.5 ± 8.5

Figure S3. Unwinding of partial duplex. (**A**) Schematic smFRET partial duplex construct before and after unwinding. (**B**) Unwinding kinetics of 18mer with T15, 18mer with T40 and 42mer with T15 (i.e. $(TTAGGG)_4$ duplex_T15) partial duplex fitted to Michaelis-Menten plot and the table contained V_{max} and K_m of the respective constructs.

SUPPLEMENTARY FIGURE 4

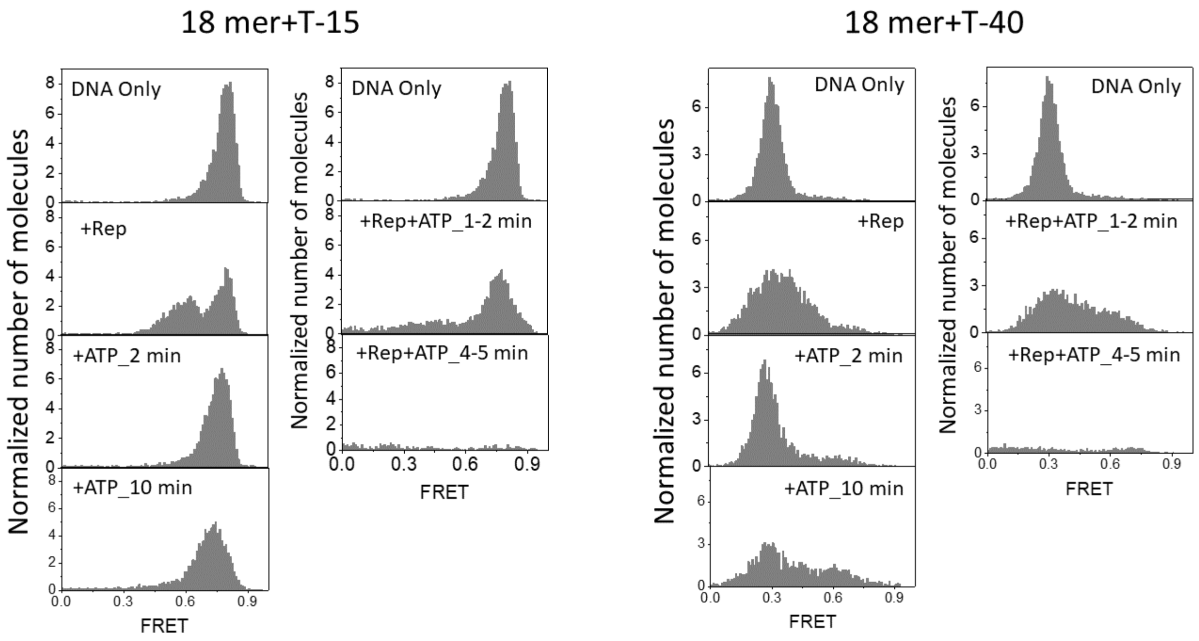

Figure S4. Rep induced partial duplex unwinding of T15 and T40 tail without G4. For each case, the left panel is protein ([Rep] = 100 nM) followed by ATP (2 mM) whereas the right panels are when Rep (100 nM) and ATP (2 mM) was added simultaneously.

SUPPLEMENTARY FIGURE 5

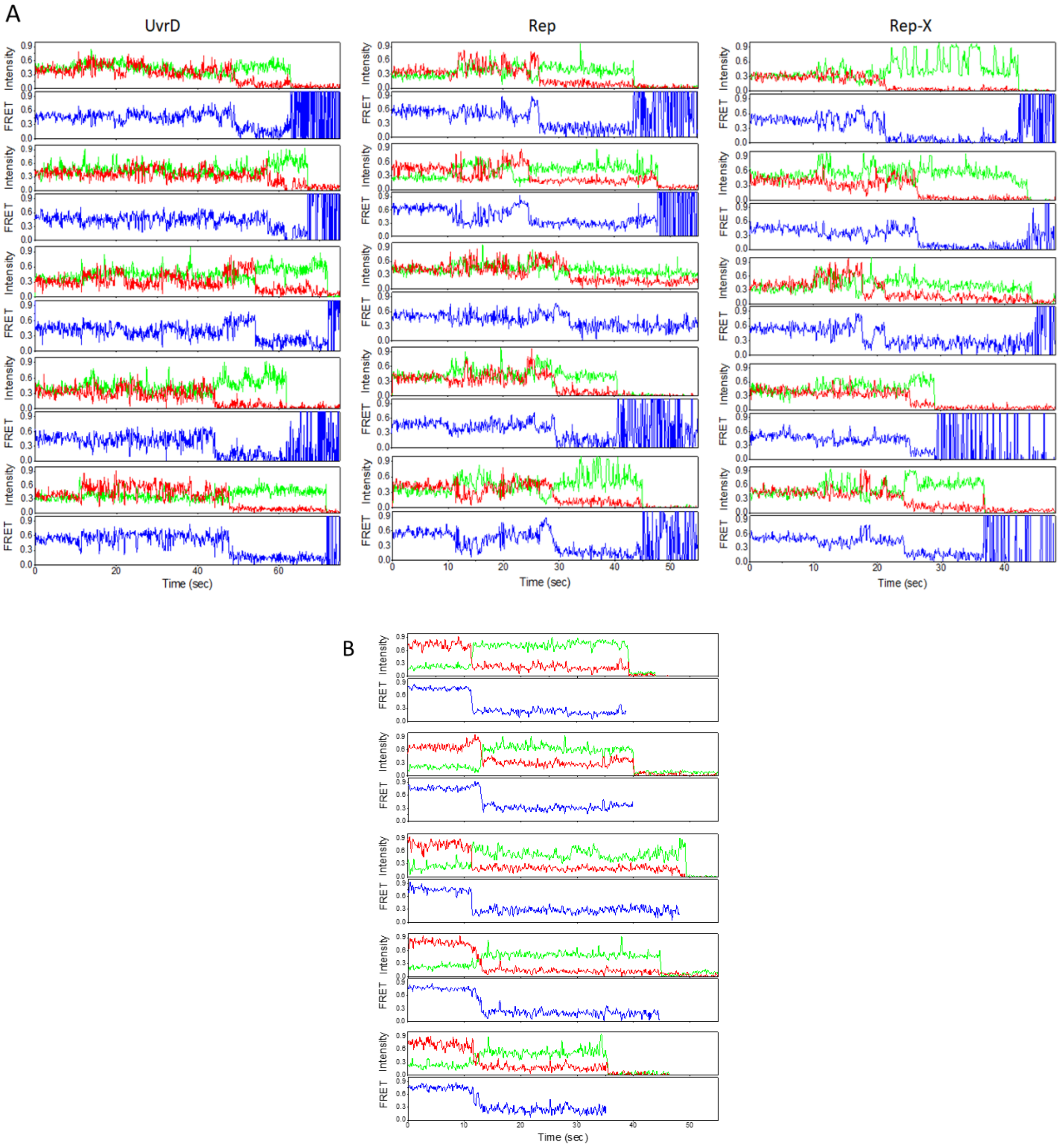

Figure S5. (A) Real time smFRET unwinding traces of G4-T15 using UvrD, Rep and Rep-X. In every cases protein added with ATP at ~10 sec. **(B)** Rep induced real time smFRET unwinding traces of T15 tail containing partial duplex (lack of G4).

SUPPLEMENTARY FIGURE 6

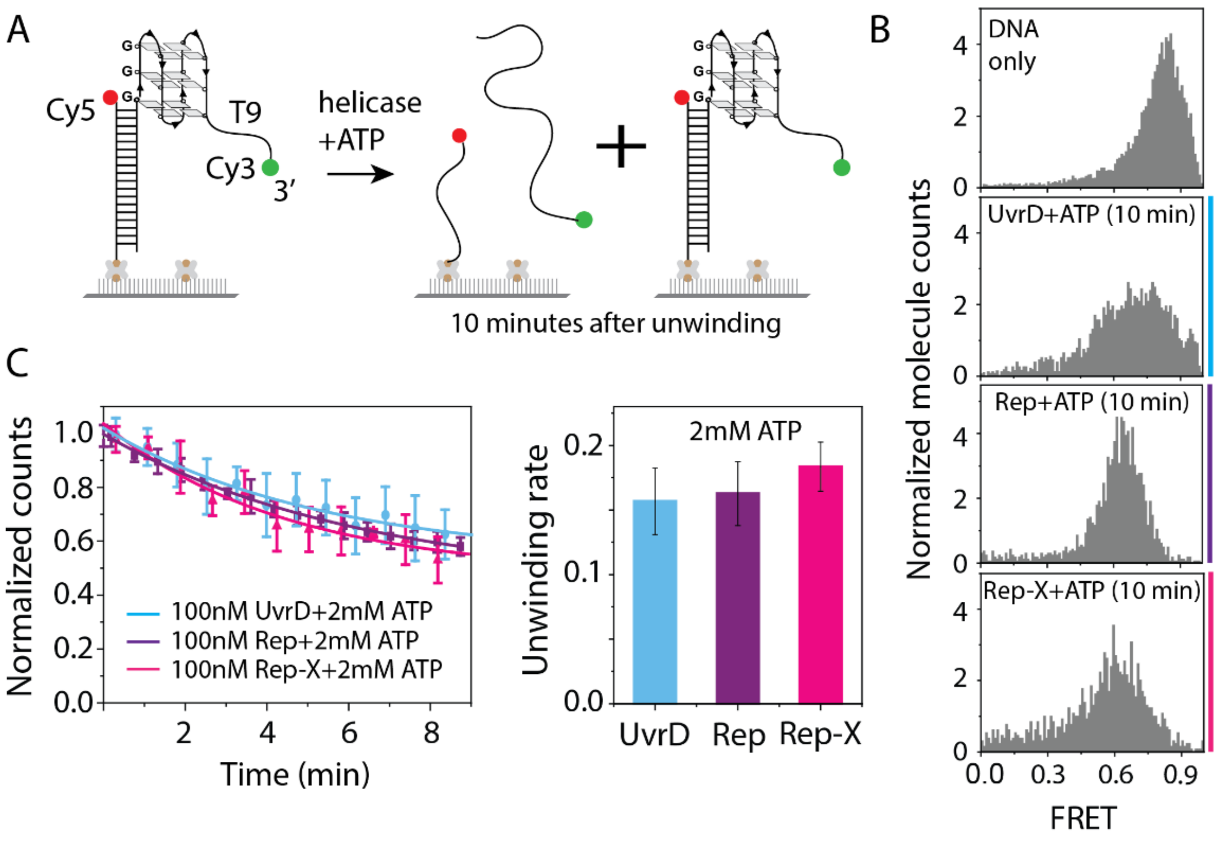

Figure S6. G4 unwinding assay with shorter tail. (**A**) Schematic smFRET diagram of helicase induced nonparallel G4 unwinding with T9 tail at 3' end. (**B**) The histogram of T9 substrate DNA only and at 10 minutes unwinding of 100 nM respective protein (UvrD, Rep and Rep-X) with 2 mM ATP. (**C**) Single exponential fitted of unwinding kinetics of UvrD, Rep and Rep-X. The bar diagram is the fitted kinetic rate.

SUPPLEMENTARY FIGURE 7

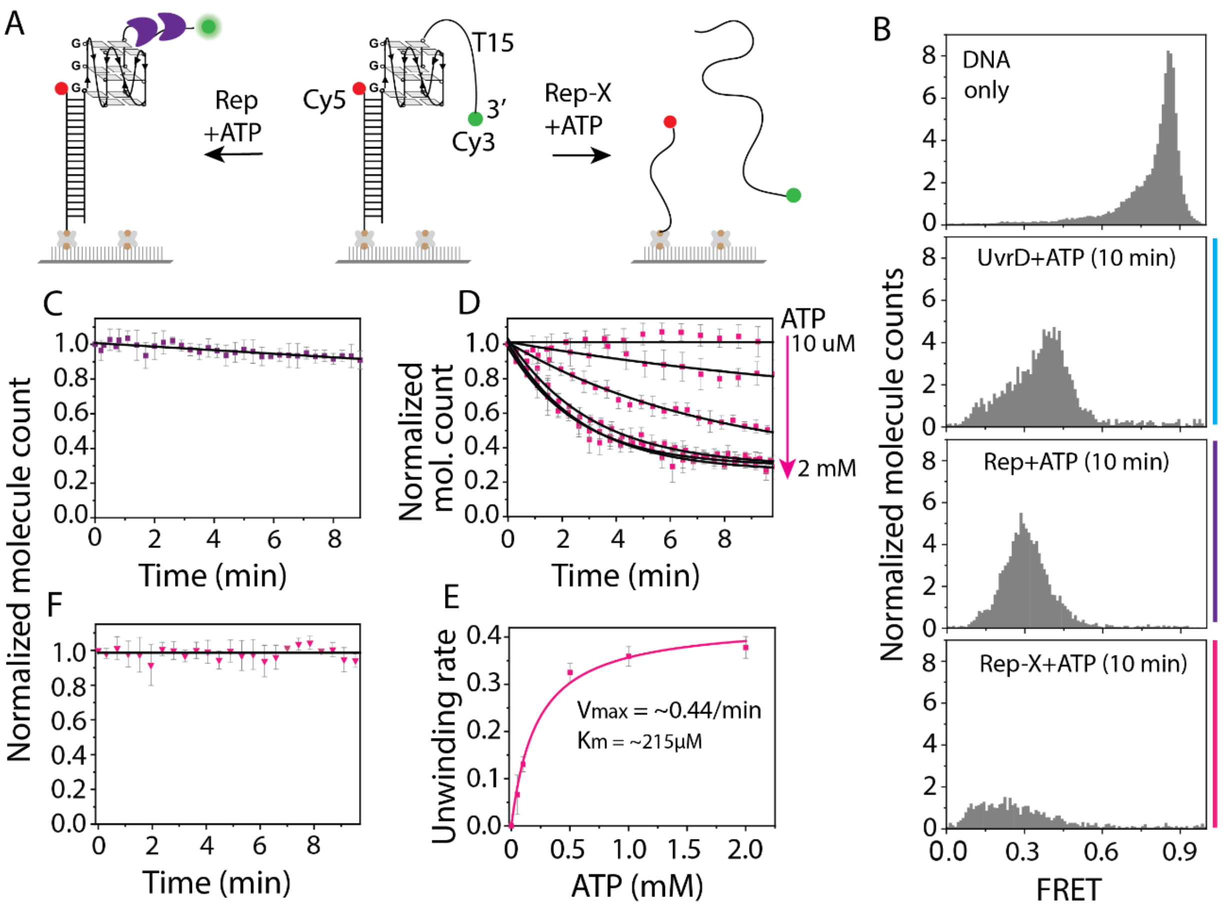

Figure S7. Unwinding of parallel G4. (**A**) Schematic smFRET model of before and after addition of helicase to the parallel G4 with T15 tail. (**B**) The histogram of DNA only and at 10 minutes unwinding with UvrD, Rep and Rep-X. (**C & D**) The unwinding kinetics of Rep (100 nM with 2 mM ATP) and Rep-X (100 nM with ATP titration). (**E**) Michaelis-Menten fitted plot of Rep-X induced unwinding. (**F**) The Rep-X induced unwinding kinetics of parallel G4 with T9 tail.

SUPPLEMENTARY FIGURE 8

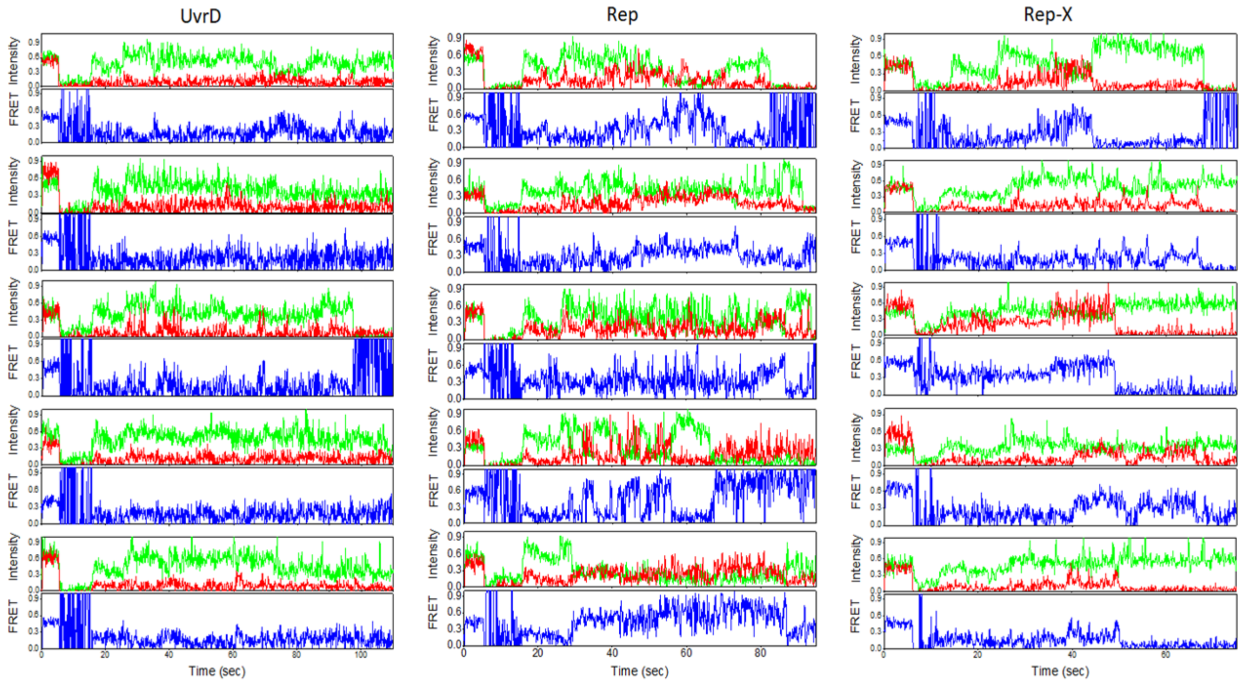

Figure S8. Real time smFRET unwinding traces of ligand bound (BRACO-19) G4-T15 using UvrD, Rep and Rep-X. In every cases ligand added at ~5 sec followed by buffer wash at ~15 sec and protein added with ATP at ~25 sec.

SUPPLEMENTARY FIGURE 9

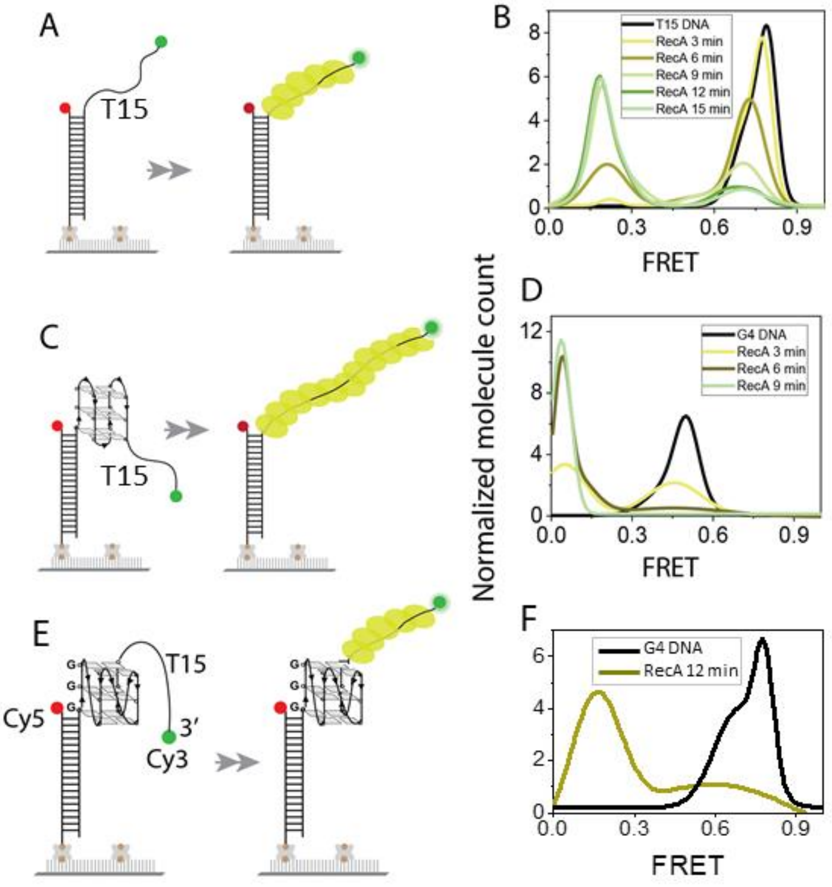

Figure S9. RecA assemble. (**A, C & E**) Schematic smFRET model of partial duplex with T15 tail, non-parallel G4-T15 and parallel G4-T15 (left panel) and the corresponding RecA filament formation (right panel). (**B, D & F**) The smFRET histogram of DNA only and with RecA (1 µM with 2 mM ATP) filament formation of corresponding DNA with time. For non-parallel G4-T15, 100 mM NaCl containing buffer was used instead of KCl. For parallel G4-T15, KCl containing buffer used and histogram suggest that RecA doesn't disrupt the G4 structure.

SUPPLEMENTARY FIGURE 10

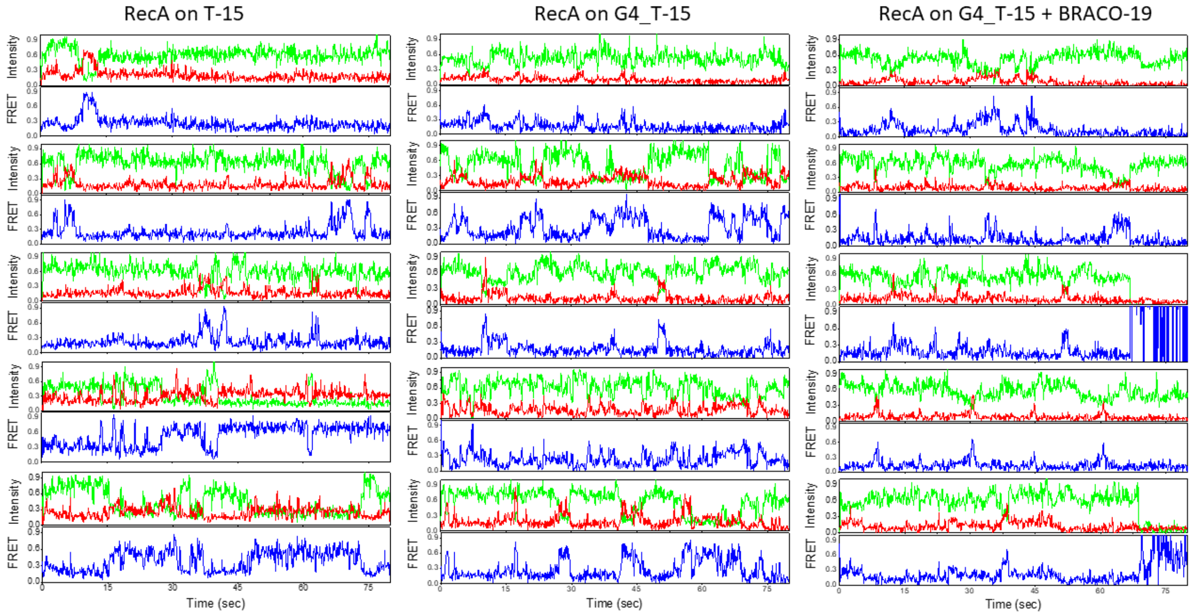

Figure S10. After RecA filament formation, the representative smFRET traces of T15, G4-T15 and after dislodge G4 ligand.
